# Synthesis and Biological Evaluation of Liguzinediol Mono- and Dual Ester Prodrugs as Promising Inotropic Agents

**DOI:** 10.3390/molecules191118057

**Published:** 2014-11-05

**Authors:** Jing Zhang, Wei Li, Hong-Mei Wen, Hao-Hao Zhu, Tian-Lin Wang, Dong Cheng, Kun-Di Yang, Yu-Qing Chen

**Affiliations:** School of Pharmacy, Nanjing University of Chinese Medicine, 138 Xianlin Road, Nanjing 210023, Jiangsu, China; E-Mails: zjczjhappy@163.com (J.Z.); njwhm@126.com (H.-M.W.); zhux58@163.com (H.-H.Z.); cpuwtl@gmail.com (T.-L.W.); dillon1987@sina.com (D.C.); aragron135@163.com (K.-D.Y.); drcoreychen@gmail.com (Y.-Q.C.)

**Keywords:** liguzinediol, prodrug, physicochemical properties, bioconversion, pharmacokinetics

## Abstract

The potent positive inotropic effect, together with the relatively low safety risk of liguzinediol (**LZDO**), relative to currently available inotropic drugs, has prompted us to intensively research and develop **LZDO** as a potent positive inotropic agent. In this study, to obtain **LZDO** alternatives for oral chronic administration, a series of long-chain fatty carboxylic mono- and dual-esters of **LZDO** were synthesized, and preliminarily evaluated for physicochemical properties and bioconversion. Enhanced lipophilic properties and decreased solubility of the prodrugs were observed as the side chain length increased. All esters showed conspicuous chemical stability in phosphate buffer (pH 7.4). Moreover, the enzymatic hydrolysis of esters in human plasma and human liver microsomes confirmed that the majority of esters were converted to **LZDO**, with release profiles that varied due to the size and structure of the side chain. *In vivo* pharmacokinetic studies following oral administration of monopivaloyl (**M5**), monodecyl (**M10**) and monododecyl (**M12**) esters demonstrated the evidently extended half-lives relative to **LZDO** dosed alone. In particular the monopivaloyl ester **M5** exhibited an optimal pharmacokinetic profile with appropriate physiochemical characteristics.

## 1. Introduction

**Figure 1:**
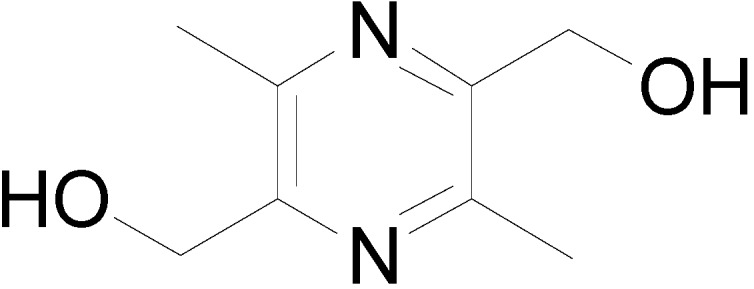
Structure of **LZDO**.

Heart failure is a complex syndrome resulting in the abnormal pumping of blood, a major cause of morbidity and mortality in cardiovascular disease [1]. Improving cardiac contractility with orally positive inotropic agents has been an important treatment of chronic heart failure. Currently orally available positive inotropic agents in the clinic primarily include digitalis, phosphodiesterase inhibitors, and dopamine. They exert positive inotropic effects in cardiac muscle by suppressing Na^+^–K^+^ ATPase, phosphodiesterase, and stimulating β-adrenergic receptor respectively [2]. However, these targets are also thought to be commonly related to ventricular arrhythmia and may lead to sudden death [3,4,5,6]. 2,5-Dihydroxymethyl-3,6-dimethylpyrazine (liguzinediol, **LZDO**, Figure 1), was found to exert markedly positive inotropic effects targeting the sarcoplasmic reticulum (SR) Ca^2+^ATPase to elevate SR Ca^2+^ transient [7]. SR Ca^2+^ATPase has been reported by Lipskaia as a potent therapeutic target for heart failure with good safety and applicability [8].

Studies in isolated hearts and diverse animal models of heart failure showed that **LZDO** has a markedly positive inotropic effect, and no arrhythmia was observed in extensive tests [9,10,11,12,13]. Further, toxicity assays proved **LZDO** highly safe, without acute and local toxicity [14], teratogenesis or mutagenicity. Its novel mechanism of action, potent positive inotropic efficacy, and relatively low safety risk have led to the preclinical development of **LZDO** as a promising patented positive inotropic agent for acute administration by injection [15,16]. The pharmacokinetic studies revealed that **LZDO** was eliminated rapidly when administrated orally or intravenously [17]. To satisfy different administration regimes in the clinic, in this work, we sought to discover novel **LZDO** derivatives as orally positive inotropic agents for chronic administration. Adequately long duration of action is clinically desirable for chronic administration, which could reduce frequent oral administrations and improve patient compliance. Based on studies of its pharmacokinetic mechanism the rapid disappearance of **LZDO** was attributed to its high polarity due to the presence of two free hydroxyl groups in the molecule [18]. Furthermore, previous structure-activity relationship studies revealed that the *para*-dihydroxylmethyl group of **LZDO** was necessary [19,20]. As stated in the literature, a prodrug might be considered when pharmacokinetically problematic moieties are essential requisites for biologically activity [21]. Hence, a solution followed to increase the half-life of **LZDO** and to offer an excellent model to deliver **LZDO** orally via masking of the alcohol hydroxyl groups through a prodrug strategy. Prodrugs are mostly pharmacologically inactive but can be activated by enzymes in body to continuously release therapeutically active metabolites, and by this means increased bioavailability, decreased metabolic inactivation, and prolonged duration of action can be achieved [22]. Nowadays, prodrugs are in a growing trend, accounting for 5%–7% of drugs approved worldwide in the early stages of drug discovery [23]. An extensively used approach for the development of prodrugs for active compound with hydroxyl functional groups is ester formation. Considerable efforts have already been previously made in this laboratory to research the diethylcarbonate ester prodrug of **LZDO** with respect to biological evaluations and pharmacokinetic studies [24]. It extended the oral half-life of **LZDO** to about 4 h, still less than satisfactory for the oral chronic administration. Also, its oil nature brings about formulation problems.

In previous literature, Simões *et al.* reported that as the chain length of the alkoxy group is extended, the stability of pyrazinoic acid ester prodrugs in human plasma and rat liver homogenate increased [25]. In this study, the same approach was applied to appropriately regulate the bioconversion of **LZDO** ester prodrug by varying their side chains to realize slow-release of **LZDO**. Therefore, ten novel long-chain carboxyl acid mono and dual esters of **LZDO** were synthesized via ester linkages between the hydroxyl group of **LZDO** and pivaloyl chloride, capric acid, lauric acid, palmitic acid, and stearic acid. The rationale behind the mono-ester design was that the remaining hydroxyl group in conjunction with long lipophilic chains may offer adequate lipophilicity, solubility, protein affinity, and subsequently good pharmacokinetics [26]. However, they have been ignored in previous work and only regarded as synthetic intermediates of the dual esters. The idea behind designing two pivaloyl esters is based on its extensive successful ester prodrug applications (*i.e.*, sulbactam pivoxil, cefcapene pivoxil and adefovir dipivoxil), and the hypothesis that the steric effect of its bulky molecular structure might produce favorable release of **LZDO** when encountering enzymes [27].

For optimization of oral **LZDO** prodrugs with prolonged half-life for the treatment of chronic heart failure, physicochemical properties and *in vitro* enzymatic bioconversion studies have been evaluated. Based upon our physicochemical and biochemical studies, monopivaloyl (**M5**), monodecyl (**M10**), and monododecyl (**M12**) esters of **LZDO** were included to further investigate the pharmacokinetics in SD rats by comparing with **LZDO** orally dosed alone. In this work, *in vitro* and *in vivo* studies demonstrated that an adequate lipophilic ester of **LZDO** could facilitate the successful prodrug delivery to prolong the duration of action of **LZDO**. In particular, the monopivaloyl derivative **M5** exhibited superior properties to other ester prodrugs.

## 2. Results and Discussion

### 2.1. Synthesis

**Scheme 1:**
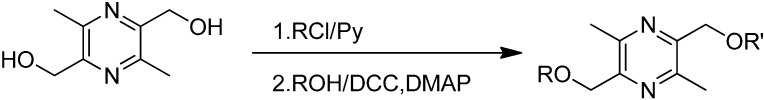
Synthesis of the prodrugs.

The reaction of **LZDO** with pivaloyl chloride in dichloromethane (DCM) solution under the catalytic action of pyridine, as conventional procedure, simultaneously resulted in compound **M5** and **D5** [28]. The remaining carboxylates were conveniently prepared in moderate yield through a one-step process by the reactions of **LZDO** with the corresponding carboxylic acids (capric acid, lauric acid, palmitic acid, and stearic acid) in DCM solution by the DMAP-catalyzed dicyclohexylcarbodiimide (DCC) method described in the literature [29]. The process is outlined in Scheme 1. Structures, melting points, yields and molecular weights of the ester products are summarized in Table 1. As shown, the melting points increased as the length of the side chain ascended in the set of linear aliphatic esters. The pivaloyl mono- and dual esters showed relatively higher melting points than corresponding linear esters. All esters synthesized here existed as solids.

**Table 1:** Structure; Yield; Melting Point; and Molecular Weight of prodrugs.

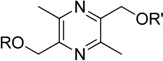					
Compound	R	R'	Yield (%)	M.p. (°C)	MW (g/mol)
**M5**	H	Pivaloyl	35	66–67	252.15
**M10**	H	*n*-Decanoyl	33	31–32	322.23
**M12**	H	*n*-Dodecanoyl	37	40–41	350.26
**M16**	H	*n*-Hexadecanoyl	30	60–62	406.32
**M18**	H	*n*-Octadecanoyl	35	65–66	434.35
**D5**	Pivaloyl	Pivaloyl	46	80–82	336.20
**D10**	*n*-Decanoyl	*n*-Decanoyl	45	46–47	476.36
**D12**	*n*-Dodecanoyl	*n*-Dodecanoyl	47	55–56	532.42
**D16**	*n*-Hexadecanoyl	*n*-Hexadecanoyl	48	74–75	644.55
**D18**	*n*-Octadecanoyl	*n*-Octadecanoyl	47	75–76	700.61

### 2.2. Physiochemical Properties

Appropriate physiochemical properties play critical roles in drug pharmacokinetics and development. The physiochemical properties including capacity factor, lipophilicity and solubility of these esters were examined and results listed in Table 2. Log *p* values were obtained by means of computation and experiment. Obviously, the aqueous solubility of **LZDO** was good, but its lipophilicity was extremely low (log *p* = −1.12), which was the primary molecular property contributing to its short half-life. After esterification, the log *p* values for prodrugs of **LZDO** ranged from 1.33 for **M5** to 3.77 for **D12** respectively. During the determination of the partition coefficients, **D16** and **D18** both thoroughly partitioned into *n*-octanol, making the log *p* undetectable and, the calculated log *p* values impossible to obtain, demonstrating that their lipophilicity was too high to be a potent drug. The results also showed that there existed good linear correlation between the calculated log *p* values and the capacity factors, suggesting that k' value could also be used to indicate lipophilicity more conveniently than the measure of log *p* values. Moreover as the carbon chain length gradually increased, the lipophilicity of the ester increased progressively, but the solubility decreased significantly. Even though the log *p* value of **D5** was comparable to that of **M10**, the solubility for the former was more than threefold higher than that of **M10**, which was postulated to result from the branching of **D5**. The lipophilicity was in the order of **LZDO** < **M5** < **D5** < **M10** < **M12** < **M16** < **M18** < **D10** < **D12**, and the solubility followed the order **M12** < **M10** < **D5** < **M5** < **LZDO**.

**Table 2:** Capacity factor, Apparent Partition Coefficient and Aqueous Solubility (*n* = 3; mean ± SD).

Compound	Capacity Factor	Log *P*^a^ (*C* log *P^b^*)	Solubility (µg/mL)
Liguzinediol	0.100 ± 0.004	−1.12 ± 0.11 (−1.49)	2.24 × 10^5^ ± 1.4 × 10^4^
**M5**	0.210 ± 0.020	1.33 ± 0.10 (0.60)	8.0 × 10^3^ ± 3.5 × 10^2^
**M10**	0.400 ± 0.015	2.48 ± 0.04 (3.60)	8.89 ± 0.21
**M12**	0.514 ± 0.012	3.12 ± 0.15 (4.66)	2.23 ± 0.11
**M16**	0.834 ± 0.012	3.25 ± 0.13 (6.77)	n.d *^c^*.
**M18**	1.055 ± 0.061	3.38 ± 0.20 (7.83)	n.d *^c^.*
**D5**	0.364 ± 0.039	2.50 ± 0.18 (2.70)	29.90 ± 1.36
**D10**	1.068 ± 0.057	2.87 ± 0.16 (8.69)	n.d *^c^.*
**D12**	1.640 ± 0.032	3.77 ± 0.21 (10.80)	n.d *^c^*.
**D16**	3.911 ± 0.042	n.d *^c^*.	n.d *^c^.*
**D18**	6.134 ± 0.084	n.d *^c^*.	n.d *^c^*.

Log *P^a^* values were carried out in 1-octanol/phosphate buffer (7.4) at 25 °C; Log *P^b^* values were calculated using ChemBioDraw Ultra 12.0 from CambridgeSoft; *^c^* n.d: Not determined.

### 2.3. Chemical Stabilities

The chemical stabilities of the target products were analyzed by high-performance liquid chromatography (HPLC) and were calculated as the amount unchanged within 24 h in buffer solutions at pH 7.4. The results are reported in Table 3. The unchanged amount of all prodrugs was virtually above 98%, indicating that all prodrugs were chemically stable.

**Table 3:** Stability of the prodrugs in buffered solutions, human serum and human liver microsomes (*n* = 3; mean ± SD).

Compound	% Unchanged at 24 h (pH 7.4) *^a^*	% Max of LZDO Released (80% Human Plasma) *^b^*	% Max of LZDO Released (Human Liver Microsomes) *^b^*	t_1/2_ (h) (80%Human Plasma) *^d^*	t_1/2_ (h) (Human Liver Microsomes) *^d^*
**M5**	98.9 ± 0.3	79.2 ± 2.2	37.5 ± 2.3	2.6 ± 0.1	5.1 ± 0.2
**M10**	98.9 ± 0.3	85.4 ± 3.1	108.1 ± 3.2	2.3 ± 0.2	2.1 ± 0.6
**M12**	99.7 ± 0.2	64.2 ± 2.3	105.1 ± 3.4	2.6 ± 0.5	2.2 ± 0.1
**M16**	99.4 ± 0.4	44.5 ± 2.2	46.3 ± 2.3	3.3 ± 0.6	3.3 ± 0.7
**M18**	98.2 ± 0.6	15.6 ± 1.6	15.9 ± 1.2	9.6 ± 1.1	9.5 ± 0.9
**D5**	96.1 ± 0.2	28.6 ± 2.4	31.3 ± 1.4	3.4 ± 0.4	3.2 ± 0.3
**D10**	98.7 ± 0.4	23.4 ± 1.2	30.9 ± 1.8	4.2 ± 0.4	4.1± 0.5
**D12**	99.8 ± 0.5	8.7 ± 1.7	8.6 ± 1.1	7.9 ± 1.0	7.9 ± 0.9
**D16**	stable *^c^*	n.d. ^e^	n.d. ^e^	n.d. ^e^	n.d. ^e^
**D18**	stable ^c^	n.d. ^e^	n.d. ^e^	n.d. ^e^	n.d. *^e^*

*^a^* Percent of compound remaining after 24 h in buffer solution; *^b^* The maximum percentage of liguzinediol released at 6 h incubation; *^c^* Compound was stable for the tested time period; *^d^* t_1/2_ values calculated from pseudo-first-order rate constant; *^e^* n.d.: Not determined due to no degradation.

### 2.4. In Vitro Metabolism

For a rationally designed prodrug intended to prolong the half-life of the active moiety, the metabolic lability should be sufficient and continuous. Preliminary metabolic stability was assessed in human plasma and human liver microsomes The profile of disappearance of each prodrug followed a pseudo-first-order trend, and half-lives were calculated from the linear slopes of logarithmic plots of remaining prodrug over time [30]. Results are listed and compared in Table 3.

The metabolic rate in human plasma and liver microsomes was quite rapid relative to that observed in buffer solution, implying that the release of **LZDO** primarily depended on enzymatic hydrolysis. In the set of linear monoesters, the maximum percentage of **LZDO** released followed the series **M10** > **M12** > **M16** > **M18**, in line with hypothesis that as the alkyl chain length extends, the metabolic stability of the ester is increased. Though **D12** could be hydrolyzed, the maximum percentage of **LZDO** released was only 8.71% in plasma and 8.60% in liver microsomes. As expected, the introduction of two pivaloyl groups on the hydroxyl groups of **LZDO** to afford **D5** weakened the production of **LZDO** relative to **M10** with similar lipophilicity, suggesting that steric effects also influenced the metabolic stability of prodrugs. The peaks of **M5**, **M10** and **M12** were observed in the HPLC chromatograms during the hydrolysis of their corresponding dual compounds, suggesting that **D5**, **D10** and **D12** produced **LZDO** via the corresponding monoesters. Consequently, the dual esters with two ester bonds to cleave released less **LZDO**. Neither any amount of **LZDO** nor the respective monoesters of **D16** and **D18** was detected, showing that there was no bioconversion in both compounds, probably due to the solubility limitation and the high hydrophobicity hindering the encounter of esterases. Normally, there existed higher expression or activity of enzymes in liver microsomes than in plasma. Interestingly, **M5** showed much higher metabolic rate in human plasma than in human liver microsomes, remarkably contrasting with other esters. Metabolic stability profiles of ester prodrugs involve a process of molecular recognition of esterase binding cavity, to which the molecular size, shape and lipophilicity of compounds were associated [31]. We therefore inferred that the steric hindrance of **M5** reduced the susceptibility of the prodrug to hydrolytic enzymes in liver microsomes more than that in plasma. In the case of prodrugs **M16**, **M18**, **D5**, **D10** and **D12**, the lack of a difference in maximum percentage of **LZDO** released in human plasma and human liver microsomes suggested that the molecular properties of these prodrugs primarily limited the liberation of **LZDO** but not the activities of the enzymes. Furthermore, the amount of **LZDO** released from **M16**, **M18**, **D5**, **D10**, **D12**, **D16** and **D18** in plasma and liver microsome was found to be poorly metabolized to permit the sufficient **LZDO** to be released. For **M5**, **M10**, and **M12**, the significantly differential maximum percentage of **LZDO** released in human plasma and human liver microsomes indicated that not only molecular properties but also activities of enzymes were involved in the liberation of **LZDO**. Moreover, the *in vitro* lability of **M5**, **M10**, and **M12** was relatively sufficient to enter into *in vivo* pharmacokinetic screening.

### 2.5. In Vivo Pharmacokinetics

**Figure 2:**
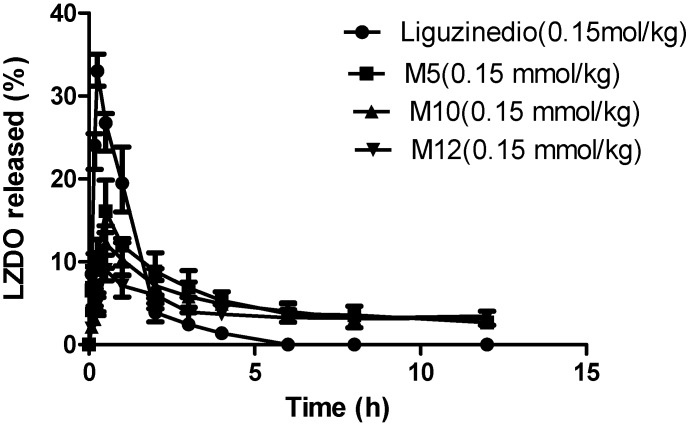
The plasma concentration *versus* time curves of **LZDO** after oral dosing of free **LZDO**, **M5**, **M10** and **M12** at equimolar doses of 0.15 mmol/kg.

The detailed pharmacokinetic parameters are reported in Table 4 and the plasma concentration *versus* time profile is depicted in Figure 2. The pharmacokinetic profiles of **LZDO** after oral dosing of three ester prodrug forms (**M5**, **M10**, **M12**) and free **LZDO** demonstrated that as the lipophilicity escalated, the plasma *C*_max_ did not increase correspondingly, but rather decreased. The plasma *C*_max_ of **LZDO** was 33 μg/mL, with **M5** being 16 μg/mL. For **M10** and **M12**, the *C*_max_ values were too low, 12.07 and 9.07 μg/mL respectively. As expected, bioavailability more or less increased after the lipophilicity of **LZDO** (log *p* = −1.12) was improved. However, it was also clear that there existed a lipophilicity threshold above which lipophilicity exerted a negative effect on AUC values. Probably, it was due to insufficient solubility of the prodrugs influencing absorption. The AUC values were in the order of **M5** > **M10** > **M12** ≈ **LZDO**. Furthermore, *T*_max_ values were 30 min with no significant difference between these three esters, a 15 min increase compared to **LZDO** dosed alone. *T*_1/2_ values of **LZDO** after prodrug administration were all longer than that of free **LZDO** administration. *T*_1/2_ of **M5** was 7.8 h, approximately an 8-fold increase compared to free oral dose of **LZDO**, a 4-fold increase relative to the diethylcarbonate prodrug of **LZDO**. For **M10** and **M12**, *T*_1/2_ was 9.99 h and 19.96 h, respectively. Focusing on half-life, the *C*_max_ and AUC values of **LZDO**, **M**5 not only exhibited evidently prolonged elimination half-life, but also relatively good pharmacokinetic features. Typically, drug metabolism involvess phase I oxidation, followed by phase II conjugation. We therefore speculated that the slower release rate of **LZDO** from **M5** in liver microsomes compared to plasma reduced the chance for phase II conjugation of **LZDO** in the liver.

**Table 4:** Pharmacokinetic parameters of **LZDO** after oral administration of free **LZDO**, **M5**, **M10** and **M12** (mean ± SD; *n* = 6).

Compound	Liguzinediol	M5	M10	M12
Dose	25.2 mg/kg (0.15 mmol/kg)	37.8 mg/kg (0.15 mmol/kg)	48.3 mg/kg (0.15 mmol/kg)	52.5 mg/kg (0.15 mmol/kg)
*T*_max_ (h)	0.25	0.50	0.50	0.50
*C*_max_ (μg·mL^−1^)	33.00 ± 1.59	16.56 ± 2.54	12.07 ± 1.69	9.07 ± 1.11
*t*_1/2_ (h)	1.06 ± 0.47	7.81 ± 5.43	9.99 ± 8.64	19.96 ± 9.96
CL/F (L·h^−1^·kg^−1^)	0.59 ± 0.04	0.45 ± 0.22	0.57 ± 0.24	0.43 ± 0.19
AUC_0–12h_ (μg·h·mL^−1^)	40.43 ± 2.26	64.58 ± 9.03	57.78 ± 3.31	49.06 ± 3.24

## 3. Experimental Section

### 3.1. Materials and Solutions

2,5-Dihydroxymethyl-3,6-dimethylpyrazine (**LZDO**, purity = 99.1%) was prepared according to published procedures in our laboratory. Pivaloyl chloride, and pyridine were purchased from Nanjing Chemical Reagent Co., Ltd (Nanjing, China). Capric acid, lauric acid, palmitic acid, stearic acid, dicyclohexylcarbodiimide (DCC), 4-dimethylaminopyridine (DMAP), and 50 mM phosphate buffer (pH 7.4) were purchased from Sinopharm Chemical Reagent Co., Ltd (Nanjing, China). Human plasma was purchased from the Nanjing Red Cross Blood Center (Nanjing, China). Human liver microsomes and β-nicotinamide adenine dinucleotide 2'-phosphate reduced tetrasodium salt (NADPH) were purchased from Sigma-Aldrich (St. Louis, MO, USA). All solvents were of analytical grade. All other reagents were of HPLC grade. Double distilled water was used in all experiments.

### 3.2. Synthesis

The control of various reactions was monitored by thin layer chromatography (TLC). Melting points were determined on a Buchi B-540 melting point apparatus. The structures of all prodrug compounds were characterized by ^1^H-NMR (Bruker AV 300, Bruker BioSpin AG, Faellanden, Switzerland), ^13^C-NMR (Bruker AV 300), IR (Nicolet-100, Thermo Fisher Scientific Inc., Waltham, MA, USA), and mass spectra (Triple TOF 5600, AB SCIEX Corp., Framingham, MA, USA), and their purities were detected to be above 98% by HPLC (Waters 2695, Waters Corp., Milford, MA, USA).

#### 3.2.1. Preparation of 3,6-Dimethyl-5-hydroxymethyl-2-pivaloyloxymethylpyrazine (**M5**) and 3,6-Dimethyl-2,5-dipivaloyloxymethylpyrazine (**D5**)

**LZDO** (1.68 g, 10 mmol) was dissolved in dichloromethane (20.0 mL, 26.5 g, 312 mmol), and the solution was placed on an ice bath. Then pivaloyl chloride (5 mL, 4.925 g, 40 mmol) and distilled pyridine (1.15 mL, 1.14 g, 14.35 mmol) were added slowly. After that, the reaction was kept for half an hour, then the ice bath was withdrawn and the mixture was stirred at ambient temperature for 12 h. The reaction process was monitored by TLC using petroleum–ethyl acetate (3:1), with R_f_ of M5 being 0.3, and D5 0.7. The mixture was washed with saturated sodium bicarbonate solution (3 × 100 mL), extracted with ethyl acetate (4 × 50 mL), dried over anhydrous sodium sulphate, filtered, and the solvent was evaporated under vacuum. The purification of the two compounds was performed twice by column chromatography, using petroleum–ethyl acetate (3:1), to yield **M5** and **D5**. The process is shown in Scheme 1.

*3,6-Dimethyl-5-hydroxymethyl-2-pivaloyloxymethylpyrazine* (**M5**). waxy white solid; mp 66–67 °C; yield 35%; ^1^H-NMR (300 MHz, CDCl_3_) δ 1.24 (9H, s), 2.44 (3H, s), 2.59 (3H, s), 4.24 (1H, s), 4.72 (2H, d, J = 3.75 Hz), 5.23 (2H, s); ^13^C-NMR (75 MHz, CDCl_3_) δ 19.26, 20.32, 27.20, 38.91, 60.97, 64.95, 146.42, 147.16, 148.22, 150.15, 178.06; IR (KBr): 3263 (υ OH), 2972 (υ^as^ CH_3_), 2872 (υ^s^ CH_3_), 1728 (υ C=O), 1429 (δ^as^ CH_3_), 1394, 1367 (δ^s^ CH_3_), 1151 (υ C-O) cm^−1^; ESI(+)-MS [M+H]^+^: calcd. for C_13_H_20_N_2_O_3_ 253.1474, found 253.1521. HPLC analysis showed a purity of 98.12%.

*3,6-Dimethyl-2,5-dipivaloyloxymethylpyrazine* (**D5**). Waxy white solid; mp 80–82 °C; yield 46%; ^1^H-NMR (300 MHz, CDCl_3_) δ 1.26 (9H × 2, s), 2.55 (3H × 2, s), 5.22 (2H × 2, s); ^13^C-NMR (75 MHz, CDCl_3_) δ 20.42, 27.23, 38.92, 64.91, 147.49, 149.20, 178.08; IR (KBr): 2982 (υ^as^ CH_3_), 2873 (8^s^ CH_3_), 1730 (υ C=O), 1421 (υ^as^ CH_3_), 1396, 1366 (δ^s^ CH_3_), 1148 (υ C-O) cm^−1^; ESI(+)-MS [M+H]^+^: calcd for C_18_H_28_N_2_O_4_ 337.2049, found 337.2118. HPLC analysis showed a purity of 98.45%.

#### 3.2.2. General Preparation Procedure for Linear Chain Esters

To a stirred solution of carboxylic acid (10 mmol) in anhydrous CH_2_Cl_2_ (20.0 mL, 26.5 g, 312 mmol), DMAP (60 mg, 0.49 mmol) and **LZDO** (1.68 g, 10 mmol) were added. DCC (2.06 g, 10 mmol) was added to the reaction mixture at 0 °C, which was then stirred for 30 min at 0 °C and the temperature was allowed to rise to room temperature, with stirring for another 8 h. The precipitate was then filtered off to obtain filtrate which was successively washed twice with 0.5 N HCl and with saturated NaHCO_3_ solution, dried over anhydrous MgSO_4_. The solvent was removed by evaporation and immediately purified by silica gel column chromatography, using petroleum–ethyl acetate (3:1) as eluent, to yield corresponding mono-ester and dual-ester from one reaction. The carboxylic acids used were capric acid, lauric acid, hexadecanoic acid and, stearic acid, respectively. The procedure is shown in Scheme 1.

*2-Decanoyloxymethyl-3,6-dimethyl-5-hydroxymethylpyrazine* (**M10**). Waxy white solid; mp 31–32 °C; yield 33%; ^1^H-NMR (300 MHz, CDCl_3_) δ 0.89 (3H, t, *J* = 6.39 Hz), 1.27–1.29 (12H, m), 1.64 (2H, m), 2.39 (2H, t, *J* = 7.41 Hz), 2.47 (3H, s), 2.60 (3H, s), 4.24 (1H, t, *J* = 4.62 Hz), 4.72 (2H, d, *J* = 4.5 Hz), 5.25 (2H, s); ^13^C-NMR (75 MHz, CDCl_3_) δ 14.07, 19.29, 20.37, 22.63, 24.94, 29.11, 29.22, 29.38, 31.83, 33.95, 34.08, 60.97, 64.58, 146.21, 147.35, 148.17, 150.31, 173.42; IR (KBr): 3445 (υOH), 2926 (υ^as^ CH_2_), 2854 (υ^s^ CH_2_), 1741 (υ C=O), 1425 (δ^as^ CH_3_), 1377 (δ^s^ CH_3_), 1157 (υ C-O) cm^−1^; ESI(+)-MS [M+H]^+^: calcd. for C_18_H_30_N_2_O_3_ 323.2256, found 323.2305. HPLC analysis showed a purity of 98.32%.

*2,5-Didecanoyloxymethyl-3,6-dimethylpyrazine* (**D10**). Waxy white solid; mp 46–47 °C; yield 45%; ^1^H-NMR (300 MHz, CDCl_3_) δ 0.89 (3H × 2, t, *J* = 6.36 Hz), 1.27–1.30 (12H × 2, m), 1.65 (2H × 2, m), 2.39 (2H × 2, t, *J* = 7.44 Hz ), 2.56 (3H × 2, s ), 5.23 (2H × 2, s); ^13^C-NMR (75 MHz, CDCl_3_) δ 14.07, 20.51, 22.63, 24.95, 29.12, 29.22, 29.38, 31.83, 32.78, 34.08, 64.58, 147.47, 149.35, 173.39; IR (KBr): 2925 (υ^as^ CH_2_), 2851 (υ^s^ CH_2_), 1741 (υ C=O), 1428 (δ^as^ CH_3_), 1385 (δ^s^ CH_3_), 1151(υ C-O), 721(ρ CH_2_) cm^−1^; ESI(+)-MS [M+H]+: calcd. for C_28_H_48_N_2_O_4_ 477.3614, found 477.3675. HPLC analysis showed a purity of 98.56%.

*3,6-Dimethyl-2-dodecanoyloxymethyl-5-hydroxymethylpyrazine* (**M12**). Waxy white solid; mp 40–41 °C; yield 37%; ^1^H-NMR (300 MHz, CDCl_3_) δ 0.88 (3H, t, *J* = 6.81 Hz), 1.21–1.40 (16H, m), 1.63 (2H, m), 2.35 (2H, t, *J* = 7.35 Hz), 2.44 (3H, s), 2.59 (3H, s), 4.25 (1H, t, *J* = 4.65 Hz), 4.75 (2H, d, *J* = 4.5 Hz), 5.20 (2H, s); ^13^C-NMR (75 MHz, CDCl_3_) δ 14.09, 19.29, 20.37, 22.67, 24.95, 29.12, 29.23, 29.34, 29.44, 29.57, 29.67, 31.91, 34.09, 60.97, 64.58, 146.21, 147.34, 148.17, 150.31, 173.42; IR (KBr): 3356 (υOH), 2918 (υ^as^ CH_2_), 2849 (υ^s^ CH_2_), 1737 (υ C=O), 1424 (δ^as^ CH_3_), 1379 (δ^s^ CH_3_), 1162 (υ C-O), 719 (ρ CH_2_) cm^−1^; ESI(+)-MS [M+H]^+^: calcd. for C_20_H_34_N_2_O_3_ 351.2569, found 351.2618. HPLC analysis showed a purity of 98.68%.

*2,5-Didodecanoyloxymethyl-3,6-dimethylpyrazine* (**D12**). Waxy white solid; mp 55–56 °C; yield 47%; ^1^H-NMR (300 MHz, CDCl_3_) δ 0.89 (3H × 2, t, *J* = 6.93 Hz), 1.25–1.40 (16H × 2, m), 1.64 (2H × 2, m), 2.39 (2H × 2, t, *J* = 7.59 Hz), 2.56 (3H × 2, s), 5.22 (2H × 2, s); ^13^C-NMR (75 MHz, CDCl_3_) δ 14.07, 20.51, 22.66, 24.95, 29.12, 29.23, 29.30, 29.42, 29.57, 31.88, 34.07, 64.58, 147.46, 149.34, 173.37; IR (KBr): 2915 (υ^as^ CH_2_), 2849 (υ^s^ CH_2_), 1738 (υ C=O), 1416 (δ^as^ CH_3_), 1381 (δ^s^ CH_3_), 1160 (υ C-O), 719 (ρ CH_2_) cm^−1^; ESI(+)-MS [M+H]^+^: calcd. for C_32_H_56_N_2_O_4_ 533.4240, found 533.4299. HPLC analysis showed a purity of 98.27%.

*3,6-Dimethyl-2-hexadecanoyloxymethyl-5-hydroxymethylpyrazine* (**M16**). Waxy white solid; mp 60–62 °C; yield 30%; ^1^H-NMR (300 MHz, CDCl_3_) δ 0.89 (3H, t, *J* = 6.93 Hz), 1.21–1.40 (24H, m), 1.66 (2H, m), 2.39 (2H, t, *J* = 7.56 Hz), 2.45 (3H, s), 2.60 (3H, s), 4.24 (1H, t, *J* = 4.65 Hz), 4.72 (2H, d, *J* = 4.62 Hz), 5.25 (2H, s); ^13^C-NMR (75 MHz, CDCl_3_) δ 14.07, 19.29, 20.35, 21.40, 21.52, 22.65, 24.87, 24.93, 29.11, 29.21, 29.29, 29.41, 29.56, 31.87, 34.02, 34.07, 60.99, 64.56, 146.21, 147.36, 148.16, 150.33, 173.39; IR (KBr): 3361 (υ OH), 2917 (υ^as^ CH_2_), 2849 (υ^s^ CH_2_), 1737 (υ C=O), 1425 (δ^as^ CH_3_), 1380 (δ^s^ CH_3_), 1162 (υ C-O), 719 (ρ CH_2_) cm^−1^; ESI(+)-MS [M+H]^+^: calcd. for C_24_H_42_N_2_O_3_ 407.3195, found 407.3231. HPLC analysis showed a purity of 98.76%.

*2,5-Dihexadecanoyloxymethyl-3,6-dimethylpyrazine* (**D16**). Waxy white solid; mp 74–75 °C; yield 48%; ^1^H-NMR (300 MHz, CDCl_3_) δ 0.90 (3H × 2, t, *J* = 6.93 Hz), 1.25–1.40 (24H × 2, m), 1.67(2H × 2, m), 2.40 (2H × 2, t, *J* = 7.56 Hz), 2.57 (3H × 2, s), 5.23 (2H × 2, s); ^13^C-NMR (75 MHz, CDCl_3_) δ 14.11, 17.67, 20.53, 22.68, 24.96, 29.14, 29.25, 29.35, 29.45, 29.59, 29.68, 31.92, 34.09, 64.59, 147.47, 149.15, 179.37; IR (KBr): 2916 (υ^as^ CH_2_), 2849 (υ^s^ CH_2_), 1738 (υ C=O), 1420 (δ^as^ CH_3_), 1381 (δ^s^ CH_3_), 1161 (υ C-O), 719 (ρ CH_2_) cm^−1^; ESI(+)-MS [M+H]^+^: calcd. for C_40_H_72_N_2_O_4_ 645.5492, found 645.5572. HPLC analysis showed a purity of 98.17%.

*3,6-Dimethyl-5-hydroxy-2-octadecanoyloxymethylpyrazine* (**M18**). Waxy white solid; mp 65–66 °C; yield 35%; ^1^H-NMR (300 MHz, CDCl_3_) δ 0.89 (3H, t, *J* = 6.99 Hz), 1.21–1.40 (28H, m), 1.66 (2H, m), 2.39 (2H, t, *J* = 7.56 Hz), 2.45 (3H, s), 2.60 (3H, s), 4.25 (1H, t, *J* = 4.56 Hz), 4.72 (2H, d, *J* = 4.56 Hz), 5.25 (2H, s); ^13^C-NMR (75 MHz, CDCl_3_) δ 14.10, 19.29, 20.38, 22.68, 24.95, 29.13, 29.24, 29.34, 29.44, 29.58, 29.68, 31.91, 34.09, 60.97, 64.58, 146.21, 147.34, 148.17, 150.31, 173.43; IR (KBr): 3411(υ OH), 2917 (υ^as^ CH_2_), 2849 (υ^s^ CH_2_), 1737 (υ C=O), 1425 (δ^as^ CH_3_), 1380 (δ^s^ CH_3_), 1163 (υ C-O), 719 (ρ CH_2_) cm^−1^; ESI(+)-MS [M+H]^+^: calcd. for C_26_H_46_N_2_O_3_ 435.3508, found 435.3542. HPLC analysis showed a purity of 98.69%.

*3,6-Dimethyl-2,5-dioctadecanoyloxymethylpyrazine* (**D18**). Waxy white solid; mp 75–76 °C; yield 47%; ^1^H-NMR (300 MHz, CDCl_3_) δ 0.90 (3H × 2, t, *J* = 6.93 Hz), 1.25–1.40 (28H × 2, m), 1.67 (2H × 2, m), 2.38 (2H × 2, t, *J* = 7.56 Hz), 2.58 (3H × 2, s), 5.25 (2H × 2, s); ^13^C-NMR (75 MHz, CDCl_3_) δ 14.11, 17.67, 20.53, 22.68, 24.96, 29.14, 29.25, 29.35, 29.45, 29.59, 29.68, 31.92, 34.09, 64.61, 147.47, 149.15, 179.37; IR (KBr): 2917 (υ^as^ CH_2_), 2848 (υ^s^ CH_2_), 1738 (υ C=O), 1425 (δ^as^ CH_3_), 1162 (υ C-O), 719 (ρCH_2_) cm^−1^; ESI(+)-MS [M+H]^+^: calcd. for C_44_H_80_N_2_O_4_ 701.6118, found 701.6191. HPLC analysis showed a purity of 98.38%.

### 3.3. Physicochemical Properties

#### 3.3.1. Determination of Lipophilic Index

According to the method described in the literature [32], the capacity factor (k') of the prodrugs was determined using high-performance liquid chromatography (HPLC). Retention times of each prodrug and LZDO were measured and k' values were calculated from the following equation:

k' = (t' − t_0_)/t_0_ (1)

where t_0_ is the retention time of the uracil and t' is the retention time of each compound. All experiments were conducted in triplicate.

#### 3.3.2. Partition Coefficient Determination

Partitioning studies were performed with *n*-octanol and 50 mM phosphate buffer solution (pH 7.4) following standard procedures described in the literature [33]. All experiments were conducted in triplicate, and the determination of **LZDO** was also carried out as a reference. Meanwhile, ChemBioDraw Ultra 12.0 was used to determine the calculated log *p* values as reference to the observed values.

#### 3.3.3. Solubility Studies

An excess of each prodrug was added to 5 mL of 50 mM phosphate buffer solution (pH 7.4), and shaken at 25 °C in a water bath to establish equilibration for 24 h. After the equilibration, the samples were filtered through a membrane filter (0.45 μm) and analyzed by HPLC. All experiments were conducted in triplicate [34].

### 3.4. Hydrolysis in Phosphate Buffer (pH 7.4)

Methanolic stock solutions of each compound were added to 10 mL of 50 mM phosphate buffer solution (pH 7.4). The resulting solution was placed in a constant shaker bath set at 37 °C and after 24 h, a 10 μL aliquot of reaction solution was taken and analyzed by HPLC [35]. All experiments were performed in triplicate.

### 3.5. Hydrolysis in Human Serum

A prodrug (100 μL, initial concentrations were 4.20–9.85 mM) in methanol was added to human plasma (1000 μL) diluted to 80% with 50 mM phosphate buffer solution (pH 7.4) and was placed in a constant shaker bath set. The resulting solution was subsequently incubated at 37 °C and at appropriate time intervals methanol (1 mL) containing caffeine as internal standard was added in order to deproteinize the serum. The sample was vortexed for 3 min, and then centrifuged for 5 min at 5000 *g*. 900 μL of clear supernatant was withdrawn and evaporated to remove the solvent. Before analysis by RP-HPLC, the samples were dissolved in methanol (200 μL), vortexed again for 5 min, and then centrifuged for 5 min at 12,000 *g*. All experiments were performed at least in triplicate.

### 3.6. Metabolism Studies in Human Liver Microsomes

To the human liver microsomes which were dissolved in 50 mM phosphate buffer solution (pH 7.4) to a concentration of 1 mg/mL, a prodrug (100 μL, initial concentrations were 4.20–9.85 mM) in methanol and NADPH (100 μL, 1 mM) also in 50 mM phosphate buffer solution (pH 7.4) were successively added to initiate the incubation. The solution was then incubated at 37 °C and the subsequent procedures of sample preparations were similar to those in Section 3.5. All experiments were performed at least in triplicate.

### 3.7. In Vivo Pharmacokinetic Studies

#### 3.7.1. Rat Experiment

The experiments were performed in the certified Laboratory Animal Center (Nanjing University of Chinese Medicine, Nanjing, China); the license number of the rats was SCXK (Zhejiang) 2014–0001. The studies were conducted according to the Guide for the Care and Use of Laboratory Animals [36]. Male Sprague-Dawley rats (Hangzhou, China) weighing 220–250 g were fasted for 12 h prior to experiments with free access to water. Animals were randomly assigned to four groups and each group received one substance (**LZDO**, **M5**, **M10**, and **M12**) suspended in 0.5% CMC-Na solution [37]. The dosages of prodrug **M5** (0.15 mmol/kg), **M10** (0.15 mmol/kg), and **M12** (0.15 mmol/kg) were 37.8 mg/kg, 48.3 mg/kg, 52.5 mg/kg, respectively. All dosages of prodrug were equivalent to 25.5 mg/kg of **LZDO** (0.15 mmol/kg). After oral administration of all substances by oral gavage, blood samples of 0.3 mL were collected from the ophthalmic venous plexus and put into heparinized Eppendorf tubes at 0.083, 0.167, 0.25, 0.5, 1, 2, 3, 4, 6, 8 and 12 h [38]. Blood samples from each animal were immediately centrifuged for 10 min at 5000 *g* and 100 μL of plasma was then removed and stored at −80 °C until determination by HPLC.

#### 3.7.2. Sample Treatment

Before analysis, blood samples were taken and thawed. Ten μL of 0.1 mg/mL caffeine as internal standard was added to blood sample. The following procedures such as precipitation, centrifugation and reconstitution were the same as sample preparations in Section 3.5.

#### 3.7.3. Pharmacokinetics and Statistical Analysis

*C*_max_ and *T*_max_ values were directly obtained from the plasma-concentration time curves and the area under the plasma-concentration time curves (AUC_0→t_) were determined by the trapezoidal method. The other pharmacokinetic parameters were calculated on the basis of non-compartmental analysis of the concentration-time profile using pharmacokinetic software package DAS.2.0. The results were expressed as mean ± SD.

### 3.8. Analytical Procedures

Separation and quantitation of the releasing **LZDO** and the prodrugs were carried out using a HPLC procedure. The HPLC system was equipped with a Waters 2695 gradient pump, a Waters 2487 UV detector at 278 nm, and an Empower 2 data station. The analytical column for *in vitro* samples was a Boston Green ODS-AQ column (4.6 mm × 50 mm, 5 μm) and the injection volume was 10 μL. Gradient elution methods were performed with a flow rate at 1.0 mL/min on Boston Green ODS-AQ column maintained at 30 °C. The percentage of mobile phase consisting of methanol and water was varied to elute analytes (see Supplementary Material for details of the HPLC elution). The methods provided rapid analysis for quantifying **LZDO**, prodrug and internal standard simultaneously. When qualifying the releasing amount of **LZDO**, separate standard curves under specific analytic condition were prepared by spiking a known amount of **LZDO** and internal standard in blank matrix. Disappearance of each prodrug from *in vitro* samples was analyzed by taking the ratio of peak areas detected at sampling time to that at initial time. As for *in vivo* pharmacokinetic analysis, determinations only referred to **LZDO** and an Ultimate XB-C18 column (3.0 mm × 50 mm, 3 μm) with an injection volume of 2 μL was used. The flow rate on Ultimate XB-C18 column was 0.4 mL/min with a column temperature of 30 °C. The mobile phase consisted of methanol (A) and water (B). The initial mobile phase composition was 4% A/96% B and maintained for 8 min. After that the mobile phase composition was changed to 80% A/20% B over 4 min and held at that ratio for an additional 4 min. The mobile phase was returned to initial composition over 1 min and re-equilibrated for 3 min. For *in vivo* analytical samples, a separate standard curve was prepared using blank rat plasma and ranged from 0.39 to 50 μg/mL.

## 4. Conclusions

For **LZDO**, the hurdle of a desirable pharmacokinetic profile for the oral chronic administration is low lipophilicity resulting in its rapid elimination. Nevertheless, *in vivo* studies also demonstrated that too poor solubility of **M10** and **M12** also impeded satisfactory pharmacokinetic characteristics, presenting very low *C*_max_ values. This study in principle observed that the **LZDO** ester with carbon atom linear chain lengths above 10 was less feasible to release **LZDO** sufficiently relative to **M5** while it increased metabolic stability and extended the retention of **LZDO**. Pivaloyl moieties are often applied in prodrug strategies to accomplish different purposes. Herein, the application of conjugating a pivaloyl moiety with **LZDO** to release **LZDO** slowly was also successful. Further, a molecular weight of 254 g/mol, adequate lipophilicity with log *p* of 1.33, and good solubility (8 mg/mL) of the monopivaloyl ester jointly contributed to a favorable pharmacokinetic profile and extended half-time, according to “Lipinski’s rule of five” [39]. During synthesis, we also found that monoester prodrugs of **LZDO** containing eight carbon atom linear chain lengths existed as oils (data not shown). Thus, a monoester prodrug of **LZDO** with a carbon atom chain length of less than 10 will be considered to be designed in the form of a branched chain; of which we would like to further explore as a promising **LZDO** ester prodrug candidate in the future.

In summary, the length and structure of the alkyl side chain in prodrug molecules determined the physiochemical properties, *in vitro* metabolic stability and the *in vivo* pharmacokinetics of **LZDO**. In other words, introducing “prodrug design” into drug design of new chemical entities in the earliest stages of research and development might help **LZDO** to overcome the deficiency of being unable to show an adequate half-life in its basic form for oral chronic medication. In this work, a monopivaloyl ester presented the optimal pharmacokinetic feature with intriguing physiochemical properties, and was selected for further development.
